# Thermal Properties of Alkali Activated Slag Plaster for Wooden Structures

**DOI:** 10.1038/s41598-020-57515-8

**Published:** 2020-01-20

**Authors:** Andrius Kielė, Danutė Vaičiukynienė, Gintautas Tamošaitis, Rėda Bistrickaitė

**Affiliations:** Kaunas University of Technology, Studentu st. 48, Kaunas, LT 51367 Lithuania

**Keywords:** Physical sciences, Engineering, Materials science, Materials for energy and catalysis

## Abstract

Currently, the production of green building materials grows up. Alkali-activated materials (AAMs) based plaster have better fire resistance properties compared to Portland cement-based concrete and plasters. Compared to Portland cement-based systems AAMs retain a significant level of structural stability after exposure to fire events. AAM based concrete doesn’t have at all or has an insignificant amount of calcium hydroxide in the binder structure which exposed to high-temperature changes to calcium oxide. This weakens Portland cement structural properties and allows cracks to appear under high-temperature conditions. This study shows that AAM based plaster that consisted of alkali-activated ground granulated blast furnace slag (slag) with the addition of Phosphogypsum (PG), sand and polypropylene fibre filling exposed to 1000 °C temperature shows up to 2% longitudinal dimension shrinkage. After exposure of elevated temperature these fibers melted leaving a network of channels that allow water vapour vaporize and inner pressure in the material decreased. The start of the wood surface charring process t_ch_ is 10 minutes after the start of heating. Using an AAM binder as fire-resistant plaster coating on a wooden structure delays the start of the char layer forming on the wood surface. This allows using AAMs base plaster for fire-resistant coatings on combustible materials as the barrier layer in order to increase the passive safety of wooden structures in heritage buildings.

## Introduction

The wood burning process has been extensively studied, but due to the uneven properties of the wood structure, the carbon layer on the wood surface and the rate of charring in the fire may vary. The rate of wood charring is highly dependent on the type, density and humidity of the wood. The prevalence of wood constructions and the properties of wood burning require additional protection to ensure the fire resistance of structures. Various protection methods and fire protection materials are used for the protection of wooden structures. Carosio *et al*.^1^ stated that the use of clay nanopaper is proposed as a fire protection coating for wood. Nine *et al*.^2^ reported a new fire-retardant which was made from graphene oxide and hydrated-sodium metaborates. Traditionally, wooden structures (walls, ceilings) are plastered. This greatly increases the longevity of use of interior and exterior surfaces of wooden buildings. This type of surface protection has been widely used in wooden architecture buildings, wooden mansions which now is a cultural heritage building in Baltic region countries. In addition, plastering building walls and ceilings created basic fire-protection for wooden structures by making them less combustion. The fire events in such buildings create not only material damage but also cultural losses.

Usually lime mortar and plaster were used, which were mostly used for plastering buildings. Exposed to high-temperature calcium hydroxide Ca(OH)_2_ in lime mortar decomposes into calcium oxide CaO and water. During this process water released from the plaster creates internal stresses and destroys plaster layer^3^. Due to the internal stresses, the explosive effect is possible at the plaster layer. When the material is heated, the free water is heated and evaporated, and in the absence of water evaporation (in the case of closed pores), high pressure is generated^4^. References^5^ indicate that the slag or ash was added to the mixture to improve the properties of the plaster. In this case plaster have better properties than Portland cement alone due to the reduction of Ca(OH)_2_ amount in the binder during exposure to high temperatures^6,7^.

A number of studies have shown that hardened AAM products have properties that improve the fire and a heat resistance of this material compared to Portland cement concrete^8–12^. AAMs have molecular network of chains of minerals linked by covalent bonds, and their chemical composition is similar to zeolites, but AAMs have a more amorphous microstructure. Such material occurs in a variety of industrial wastes such as fly ash, blast furnace slag and petroleum cracking catalytic waste and is greener than conventional Portland cement, not only it produces less CO_2_ into the environment but can also be used as a replacement for Portland cement. Vaičiukynienė *et al*.^13^ investigated the AAM cement mixture from alkali-activated slag with phosphogypsum additive. Samples of alkali-activated slag were tested at both room and high temperature. Pan *et al*.^14^ reported that the compressive strength of alkali-activated binders depends on the various phase transformations at elevated temperatures. Alkali-activated lightweight mortars which were made from fly ash with perlite and H_2_O_2_ could be used for passive fire protection systems for steel elements as reported by Carabba *et al*.^15^. Krivenko *et al*.^16^ stated that zeolite-like hydration products formed during the hardening process in AAMs and this mineral composition is suitable for a fire-resistant coating.

Referring to the literature^8,13,14^, alkali-activated slag binders have resistance to high temperatures and to fire. These binders retain their mechanical properties even at high temperatures. The cured alkali-activated slag binder has a Portland cement-like composition but a much lower amount of calcium hydroxide Ca(OH)_2_.

This paper describes the mechanical properties of the alkali-activated slag binder and sand filler mixture reinforced with polypropylene fiber. This mixture was applied on the wooden 100 × 250 × 25 mm plank surface as fire protective layer. Such construction was heated in a directional oven to simulate the fire situation then heat effects one side of the wall. The objective is to measure time before construction fails or cracks appear in protective layer. During the test temperature was measured on the unheated surface of wooden plank. After cracks and burned areas were visible on the unheated surface of the specimen the test was stopped, the specimen removed from the furnace and cold down. After what protective plaster layer was removed and formed char layer measured. Wood charring rate was calculated from dividing the thickness of formed char layer to heating time. this calculated charring rate was compared to unprotected wood charring rate.

## Materials and Methods

### Investigation methods

The dilatometry method was used for determination of the thermal shrinkage coefficient of the plaster specimens made from AAMs, sand and fibre mixture. The thermal shrinkage of these specimens is characterized by a linear shrinkage coefficient (α). The coefficient of shrinkage of solid material depends on temperature and this dependency sometimes is very complex. The average coefficient of shrinkage is most commonly used, and the shrinkage coefficient value is assumed to be constant over the temperature range.

**Figure 1:**
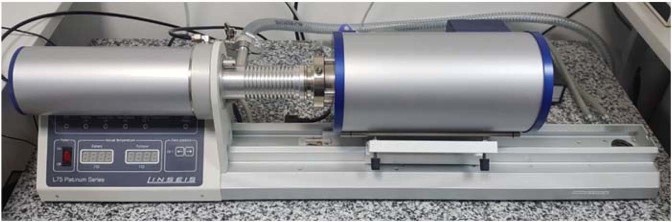
LINSEIS “LH75 PT1600” horizontal dilatometer (author photo).

The thermal shrinkage coefficient is the ratio of the elongation and the initial length of the specimen by raising the temperature at one degree^17^. The thermal shrinkage of the material is determined with a horizontal dilatometer (Fig. 1).

The hardening and compression strength of the plaster was determined according to standard LST EN 1015-11. This standard describes the method for determining the strength of bending and compression of plaster samples. The essence of the method is that the strength of the plaster during bending is determined by loading the hardened plaster prism formed at three points to breakage. The compression strength of the plaster is determined by testing two parts of the prism obtained by bending strength^18^. The compressive and flexural strength of specimens was tested using a hydraulic press ToniTechnik 2020 after 7 and 28 days. A total of six prisms were tested for each data point. The compressive strength of specimens was determined according to standard EN 196-1.

**Figure 2:**
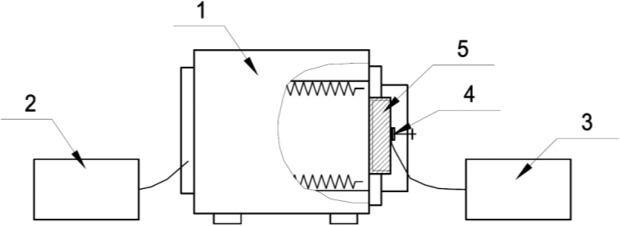
The schematic diagram of one-side heating of construction specimens: 1 - heating device; 2 – automatic thermoregulator for regulating the temperature of the heating chamber; 3 - temperature measuring equipment; 4 –thermocouple; 5 – construction specimen (author’s shema).

The one-sided heating in the furnace properties of specimens was determined according to LST EN 1363-1: 2012. In order to determine the peculiarities of plastered wood at evaluated temperatures, a special one-sided heating chamber was used according to sources^19,20^. This equipment can simulate the one-sided heating of the test sample from 20 °C to 950 °C. The one-sided heating device consisted: a thermostat, a measuring instrument and a readout device as shown in (Fig. 2)^21^.

**Figure 3:**
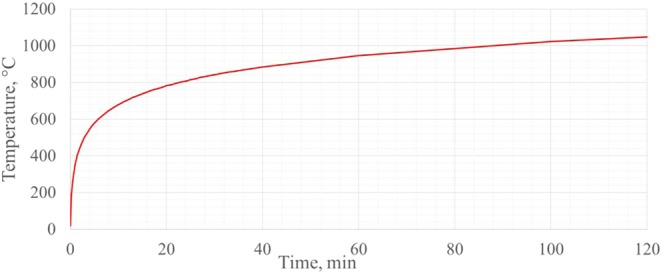
Logarithmic time and fire temperature curve^22^.

The principle of the experiment is to heat the specimen from one side to the other, as stipulated in the standard LST EN 1363 - 1: 2012 according to the temperature-time dependence -*T*. LST EN 1363-1: 2012 Fire resistance tests. Part 1. General requirements refer to the most frequent occurrence of a fire called “Nominal fire”. Automatic thermoregulator is programmed to adjust the heat rate following “Nominal fire” temperature-time dependence. During this fire event, the temperature change in the test furnace is defined by the logarithmic curve (Fig. 3)^22^.

1 $$T=345\,\log \,10(8t+1)+20$$

where: *T* - average oven temperature, in degrees Celsius; *t* - time in minutes.

The equipment described above for the test (Fig. 2) ensured the selected thermal load. Of course, the thermal distribution of the structure and the variation of the load value depending on the heating mode also influence the distribution of the temperature in the cross section of the structure^21^. Rapidly high temperature simulates the most unfavourable environmental conditions for the test substance when it starts a continuous heat transfer process that affects the physicochemical transitions in the test substance. Using said equipment (Fig. 2), the wood specimen is heated for a predetermined period of time t, min. After this time, the specimen is withdrawn from the chamber and extinguished by covering it with a non-flammable cloth or water using a spray gun^21,23^. After the specimen has cooled, it is cut through the axis of the specimen and the depth of char depth of the test specimen is determined. The slider is used to measure the remaining part of a healthy wood, and the measurement result is an average of at least 3 measurements. The thickness of the char layer of H, mm is calculated from the original specimen high subtract uncharred sample height. From the thickness of the char layer and the heating time is calculated the wood charring rate β, mm/min^21,23^.

The X-ray diffraction (XRD) analysis was performed using a D8 Advance diffractometer (Bruker AXS, Karlsruhe, Germany) operating at a tube voltage of 40 kV and tube current of 40 mA. The X-ray beam was filtered with 0.02-mm Ni filter to select the CuKa wavelength. The power X-ray diffraction patterns were identified with references available in the PDF-2 data base (PDF–2 International Centre for Diffraction Data, 12 Campus Boulevard Newtown Square, PA 19073-3273 USA). The X -ray fluorescence analysis (XRFA) of slag was performed using a fluorescence spectrometer S8 Tiger (Bruker AXS, Karlsruhe, Germany) operating at the counter gas Helium 2 bar. The hydration water in gypsum (loss on ignition, %) was calculated after heating the material at the temperature of 400 °C.

The optical microscopy images of alkali-activated slag plaster microstructures were evaluated by a CETI (Belgium) optical microscope.

### Initial materials and the composition of specimens

**Figure 4:**
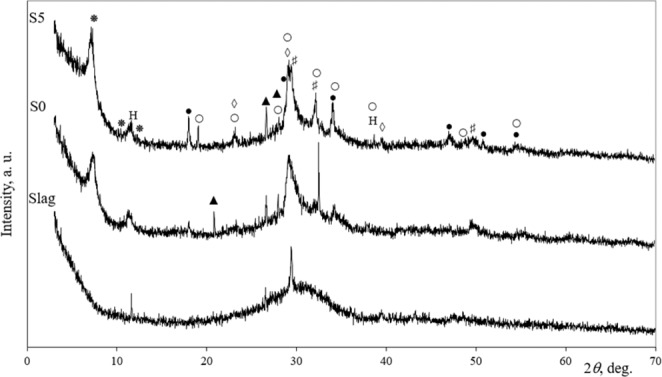
The XRD diffraction patterns of slag and alkali activated slag without PG (S0) and alkali activated slag with 5% of PG (S5). Note: were H – hydrotalcite Mg_6_Al_2_CO_3_(OH)_16_∙4H_2_O (14–191), ▲ – quartz SiO_2_ (78–2315), ◇ – calcite CaCO_3_ (72–1651), ● – portlandite Ca(OH)_2_, ○ – sodium sulfate Na_2_SO_4_, * – calcium aluminum silicate hydrate Ca_5.57_ Al_12.3_ Si_12_ O_49.2_ H_2.34_, *#* – calcium silicate hydrate Ca_1.5_ Si_3.5_ O_3.5_ xH_2_O.

In this study milled blast furnace slag was used as aluminosilicate precursor for alkali activated material. The oxide composition of the slag was given in Table 1. In this slag varied CaO and SiO_2_ oxides according to the data of XRF analysis. The mineral composition was evaluated by using XRD analysis (Fig. 4). It showed the peaks of quartz, hydrotalcite and calcite. The presence of amorphous SiO_2_ is identified as “a hill” on XRD graphic within 2θ degree in range 20°–35°. High amounts of amorphous SiO_2_ and Al_2_O_3_ make slag a good raw material for the production of alkali-activated binding material.

**Table 1:** Oxide composition of the slag and phosphogypsum %.

Oxide	CaO	SiO_2_	Al_2_O_3_	MgO	SO_3_	Na_2_O	Fe_2_O_3_	P_2_O_5_	K_2_O	F	L.I.*	Other
Slag	45.20	37.10	6.44	5.76	1.85	1.02	0.79	0.68	0.52	—	—	0.64
PG	38.86	0.30	—	0.22	52.51	—	0.04	1.61	—	0.06	6.40	—

*L. I. - the loss on ignition (400 °C).

The addition (5%) of semi-hydrate phosphogypsum (PG) generated by fertilizers plant in wet-process phosphoric acid production was used. The CaO and SO_3_ consisted of the highest amount in the PG. In this material, calcium sulphate was 92.3 wt%, according to XRF analysis (Table 1). The loss on ignition (400 °C) of this PG was 6.4%. According to the Blaine analysis results, specific surfaces areas were similar for both materials: for PG was 201 m^2^/kg and for slag was 207 m^2^/kg. Sodium hydroxide (NaOH) as alkali activator was used in the complex binder systems with slag and the addition of PG.

According to the XRD diffraction pattern in alkali activated slag without PG (S0) and with 5% of PG these crystalline components portlandite, quartz, calcite, calcium aluminum silicate hydrate hydrotalcite and calcium silicate hydrate were detected (Fig. 4). In that case when the phosphogypsum additive was used beside higher amount of portalndite Na_2_SO_4_ formed during the hydration process. Na_2_SO_4_ is well known effective activator for geopolymers or alkali activated materials^24^. The incorporation of PG in the alkali activated slag system changed the kinetic of reaction and promoted the formation of CSH in the alkali-activated slag system. The amorphous phase confirmed broad bands in the range of 25–37^o^ (2*θ*) and it area slightly increased by using PG (S5).

Sand and polypropylene fibre were used for the production of alkali-activated slag composites. The sand particles size varied from 0 mm to 2 mm, its density ρ_p_ was 1609.4 kg/m^3^ and the water content was 4.5%. Sand is a sedimentary rock with a rather diverse mineral composition, mostly quartz.

Polypropylene fibres surface area was 725 m^2^/kg ensures high mixture adhesion. The fibre is acid and alkali resistant. The amount of fibre is 1 kg/m^3^ which used in the plaster mixture.

Pinewood was used for the preparation of the wood specimen. Strength class - C24. The average density of wood is ρ_m_ - 485.2 kg/m^3^. Timber defects and branches were deselected in the production of wooden samples. The wood specimens were dried until the moisture content of the specimens did not exceed 9–10%. The moisture content of the wood specimens was measured with the HPM 20 humidity measuring device.

The strength of the hardened plaster was determined according to standard LST EN 1015-11. The bending test was performed with 160 × 40 × 40 mm samples. The bends for the bending test were made of three composite mixtures as shown in Table 2. The composition of the mixtures varies in the amount of sand filler in the mixture.

**Table 2:** The initial of mixtures of alkali-activated slag and sand filler.

Material	The ratios of slag + PG and sand		
	1.0/0.5	1.0/1.0	1.0/2.0
Milled slag, g	880.24	660.18	440.12
Phosphogypsum, g	46.32	34.74	23.16
Sodium hydroxide, g	90.00	67.50	45.00
Polypropylene fibre, g	0.50	0.50	0.50
Sand 0–2 fr., g	463.28	694.9	925.60
Water, g	290.00	217.50	170.00

## Results and Discussion

**Figure 5:**
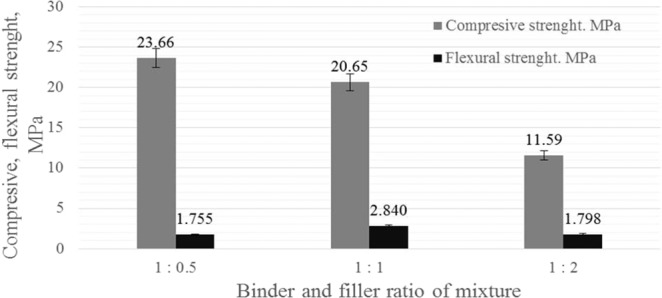
Compressive and flexural strength of alkali activated slag plasters after 28 days.

The compressive and flexural strength results of alkali-activated slag binder, sand and polypropylene fibre is showed in Fig. 5. The specimens with the lowest amount of the sand (1.0/0.5) reached the highest compression strength after 28 days (23.65 MPa). By decreasing the amount of alkali-activated slag with PG binding material (slag + PG and sand and ratios 1.0/1.0 and 1.0/2.0), the compressive strength was lower 13% and 51%, respectively by comparing with the specimens were binder and filler ratios was used 1.0/0.5. As expected to decreasing the amount of slag with PG and the increasing amount of the sand consistently reduced the compressive strength of the specimens.

The flexural strength of the (1.0/1.0) mixture samples reached the maximum bending load. With parts of (1.0/0.5) and (1.0/2.0) alkali-activated slag with PG binding material, the bending strength was 38% and 37% lower, respectively as (1.0/1.0). According to our previous investigations^13^, the specimens exhibited higher residual strength at elevated temperatures and these specimens had higher strength at ambient temperature. In addition mixture with (1.0/1.0) showed the best mechanical properties on the wooden surface. The mixture was strong enough and adhered to the wooden surface. For that reason, the this type of specimens with the lower amount of sand (1.0/1.0) was chosen for the plaster production which was applied on the wooden surface for the later measurement.

**Figure 6:**
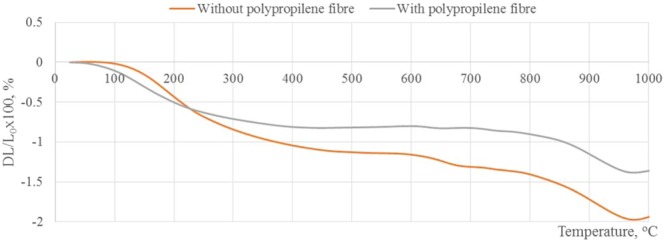
Relative linear shrinkage of the alkali activated slag plasters during dilatometry analyses.

In the next part of work the influence of elevated temperature on alkali-activated slag without polypropylene fibre and with it was investigated. The thermal behaviour of alkali-activated slag was determined by dilatometric analysis up to 1000 °C (Fig. 6). It showed changes in specimens’ dimension with increasing temperature. Both curves had a similar character. The change in shrinkage of the alkali-activated slag plaster specimens was consistent throughout the all heating period. The water gradually released from specimens and for that reason they shrink. In the event of fire plaster can collapse due to water pressure caused by evaporation of water from its rapidly rising temperature. However, in the plaster, the polypropylene fibre softens at 160 °C temperature and melts at 590 °C temperature by creating micro-channels in areas exposed to heat^25^.

The final shrinkage (at 1000 °C) was lower in the specimen with polypropylene fibre because fibre formed micro-channels for the release of water vapour. In this case, the water vapour could release from material by using these micro-channels. As a result, even after the evaporation of largest amount of water vapour, material becomes more stable and had lower shrinkage (−1.360%) compared with the specimen without polypropylene fibres shrinkage (−1.941%). This lower shrinkage could be related to denser structure^26^.

**Figure 7:**
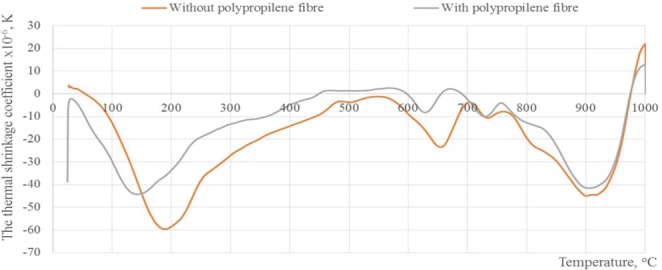
The thermal shrinkage coefficient of alkali activated slag plasters without polypropylene fibre and with it.

The thermal shrinkage coefficient curves of plasters are shown in Fig. 7. The first peak of the thermal shrinkage coefficient at 190 °C temperature could be related to the free water and water loss associated with the hydration compounds in the C-S-H type gel and alumosilicate gel^27^. The second peaks are identified around 600 to 800 °C was associated with the decomposition of carbonates: CaCO_3_ and hydrotalcite^28,29^. It should be noted that the first and second peaks of plaster with polypropylene fibre shift to lower temperatures 140 °C and 630 °C then the plaster without polypropylene fibre. This displacement of the first and second peaks can be the result of dimensional changes of the tested material when the fibre-formed micro-channels appear in the material to release water vapour when heating temperature is less than 600 °C. This is due to the thermal properties of the polypropylene fibre. The endothermic peaks located at ∼900 °C which may be due to the crystallization of different amorphous phase^30^.

**Figure 8:**
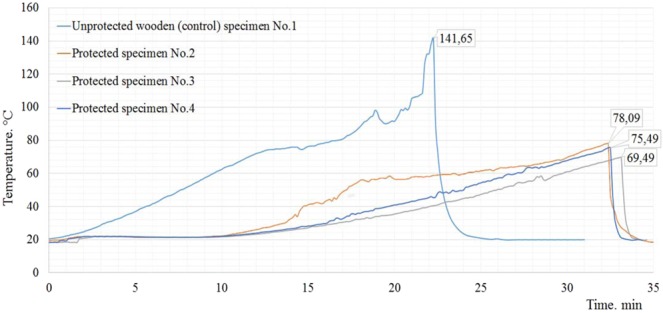
The outside temperature of plasters measured by thermocouple in recording device. Notes: No.1 is unprotected wooden specimen; No.2, No.3 and No.4 is protected with alkali-activated plaster wooden specimens.

During the one-sided heated test, two types of specimens were used. The first types of specimens were unprotected wooden planks (No 1) with 25 mm of thickness. Another type of specimens (No 2, No 3 and No 4) was protected with the layer of alkali-activated plaster. Alkali-activated plaster layer applied on planks and it was 5 mm thickness. During the first type of wood specimens exposed to elevated temperatures in the set mode until the temperature of the external thermocouple rose to 141.65 °C. The oven chamber measured 795.6 °C temperature at that time. The results of heating are shown in (Fig. 8). During the test, wood cracks were observed through the specimens and the test was terminated after 22 minutes. The wooden boards, as well as the control samples, were covered with 5 mm thick alkali-activated plaster.

The wooden planks, as well as the control samples, were covered with 5 mm thick alkali-activated plaster. These specimens were made using wooden planks, same size, thickness and properties as control specimen, and in addition, 5 mm thick layer of alkali-activated plaster was applied on one side of the planks. To acquire the similarity of the variation in heating results three specimens were tested. These specimens were made in exactly the same condition. The specimen was neatly fixed to the one-sided heating furnace which was programmed to control heating temperature during the heating time as the test conditions require. Each specimen was exposed to elevated temperatures until specimen failed to withstand heating and defect or charred crack occurred on the unheated side of specimen. The results of heating are shown in (Fig. 8). The temperature of the unheated side of the unprotected wooden sample began to rise after 1–2 minutes. Meanwhile, at that time, the furnace temperature reached 300 °C. At this temperature, the water in the wood is completely evaporated and the pyrolysis process begins on the wooden surface. The temperature curve rises at a constant rate until the cracks at the edges of the wood sample are visible and the outside temperature reaches 141.65 °C. During the unprotected wooden specimen test, cracks were observed through the specimens and the test was terminated after 22 minutes.

During the heating of the specimens with a protective layer, the test procedure was used the same as the control specimen. In all three tests, an increase in the outside temperature has been delayed and lower. It is visible from the temperature curve that the exterior temperature of the specimens begins to rise after 10 minutes from the start of the test. At the beginning of the heating, free water had to be removed from the plaster and polypropylene fibre started to melt. The melting point of the polypropylene fibre is 160 °C and the combustion temperature is 590 °C. After 8–10 minutes the temperature was reached at 600 °C inside of the oven. At this temperature, the polypropylene fibre had to melt completely and start to burn. According to the combustion temperature of the polypropylene fibre and the exterior temperature of the specimen determined during the test, it can be assumed that the start of the wood surface layer charring process t_ch_ is 10 minutes after the start of heating.

**Figure 9:**
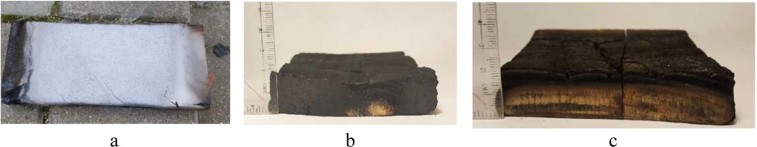
A wooden of specimen after heating experiment. Notes: (**a**) – wooden specimen with the layer of plaster, (**b**) – the heating experiment was done for specimen without alkali-activated slag plaster, and (**c**) - the heating experiment was done for specimen with alkali-activated slag plaster.

The average temperature of the external thermocouple of three tested specimen rose to 74.3 °C. The average temperature in the heating furnace chamber was then measured at 856.0 °C. Heating results are shown in Fig. 9. During the test, wood cracks were observed through the sample protective material and the wooden layer after what the test was terminated. Average time after cracks and burn defect were observed in unheated side of the specimens was 33 min.

Removing the specimen from the furnace, the plaster layer glowed hot and wooden layer tried to combust. The specimens were instantly cooled after what the plaster layer diffuses from the specimens due to a sudden change in temperature. Without cooling, the process of charring of the specimens would not stop. The wooden specimens underwent a layer of plaster but did not catch fire (Fig. 9a).

After the test, the cladding layer was cleaned, and the remaining intact wood layer measured. Depending on the dimensions of the specimens, the average minimum value of the intact wood layer is calculated. The deepest damage of specimens was 15 mm (Fig. 9b). Significant lower damage of specimens was detected for the specimen with alkali-activated slag plaster (Fig. 9c) and in this case the depth of the damage layer was about 4–5 mm.

**Figure 10:**
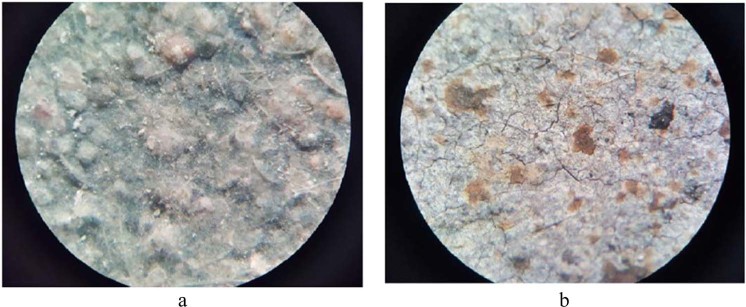
The images of alkali activated slag plaster: before (**a**) and after exposed to elevated temperatures. (**b**) The magnification 10 times.

Visual examination of the activated slag plaster after temperature exposure revealed that as the temperatures increased, the structure of specimen become stable and any the cracks had detected. The influence of elevated temperature on the changes of plaster microstructure was shown in Fig. 10. On the surface of alkaline activated slag binder, the net of polypropylene fibre and sand filler particles could be detected (Fig. 10a). After exposed to elevated temperatures the molten polypropylene fibre and the channels created in place of the fibre could be detected on the surface of the plaster (Fig. 10b). There are some micro-cracks which are caused by the evaporation of water during heating.

**Figure 11:**
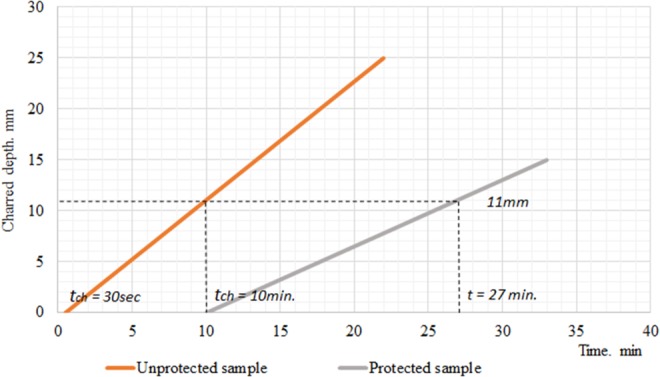
The dependance of charred depth (mm) of the wooden specimen on test time (min). Note t_ch_ the start of charring.

When comparing the protected and unprotected specimen in Fig. 11, it is noted that the plaster layer has an effect on the wood charring rate. The charred depth and the experiment time (Fig. 11) of plastered wood specimen differs from the control (unprotected) wooden specimen.

The cross-section of the unprotected specimen was completely charred due to the pyrolysis process. Cracks and defects occurred in the surface of the specimen. The exact depth of the specimen has not been determined precisely because the entire cross-section of the specimen has warped. Using averaged results, the depth of char layer was acquired and charring rate βn - 1.04 mm/min were calculated. The wood charring rate of the unprotected specimens vary from standard wood charring rate due to small dimensions and wood moisture of the specimen. The moisture content of wood has a significant influence on the rate of wood charring.

Calculated protected wood charring rate, starting from the beginning of wood surface charring t_ch_ (10 minutes) β - 0.65 mm/min. This corresponds to the very slow rate of wood charring described in experimental tests^18^.

Wooden structures are widely used in construction, but their flammability is one of the biggest negative features. Wood density, humidity, and species have a significant impact on the wood charring rate.

The balance between the thermal and mechanical properties of the wood indicates that these materials need to be additionally protected from high temperature in the case of fire using passive insulation materials which could be mechanically attached to the wooden structure.

## Conclusion

- By comparing the exterior temperature of the specimen, it is noted that the protective plaster has an impact on the wood charring rate. In all three tests, an increase in the outside temperature has been delayed. It is evident that this temperature increased after 10 minutes from the start of the test. At the beginning of the heating, free water had to be removed from the plaster and polypropylene fibre started to melt.
- The start of the wood surface layer charring process t_ch_ is 10 minutes after the start of heating. Micro-cracks in the net that occurred during the test allowed to remove water vapour and flammable gas from the wood surface. Due to this feature and plaster reinforcement, the plaster does not disintegrate throughout the heating time. This plaster did not allow the wood surface to ignite spontaneously.
- The shrinkage in length of alkali-activated slag plaster specimen with polypropylene fibre was 1.36% (at 1001 °C temperature). Meanwhile the maximal shrinkage of the refractory protective plasters is limited to −1.5% (at 1000 °C temperature). So, it is concluded that the alkali-activated slag plaster is suitable for use as a protective coating, but there should use polypropylene fibre that improve**s** the thermal properties of the plaster and decreases the final shrinkage of specimen.
- Refractory protective coatings limit the combustion of the wood surface and slow down the rate of wood charring. The alkali-activated slag binder can be used for the production of fire-resistant coatings due to its thermal shrinkage properties.
